# Expression of a protein involved in bone resorption, Dkk1, is activated by HTLV-1 bZIP factor through its activation domain

**DOI:** 10.1186/1742-4690-7-61

**Published:** 2010-07-23

**Authors:** Nicholas Polakowski, Heather Gregory, Jean-Michel Mesnard, Isabelle Lemasson

**Affiliations:** 1 East Carolina University, Department of Microbiology and Immunology, Brody School of Medicine, Greenville, NC, 27834, USA; 2 Université Montpellier 1, Centre d'Études d'Agents Pathogènes et Biotechnologies pour la Santé (CPBS), CNRS/UM1/UM2 UMR 5236, Montpellier, France

## Abstract

**Background:**

Human T-cell leukemia virus type 1 (HTLV-1) is the etiologic agent of adult T-cell leukemia, a malignancy characterized by uncontrolled proliferation of virally-infected CD4+ T-cells. Hypercalcemia and bone lesions due to osteoclast-mediated bone resorption are frequently associated with more aggressive forms of the disease. The HTLV-1 provirus contains a unique antisense gene that expresses HTLV-1 basic leucine zipper (bZIP) factor (HBZ). HBZ is localized to the nucleus where it regulates levels of transcription by binding to certain cellular transcriptional regulators. Among its protein targets, HBZ forms a stable complex with the homologous cellular coactivators, p300 and CBP, which is modulated through two N-terminal LXXLL motifs in the viral protein and the conserved KIX domain in the coactivators.

**Results:**

To determine the effects of these interactions on transcription, we performed a preliminary microarray analysis, comparing levels of gene expression in cells with wild-type HBZ versus cells with HBZ mutated in its LXXLL motifs. *DKK1*, which encodes the secreted Wnt signaling inhibitor, Dickkopf-1 (Dkk1), was confirmed to be transcriptionally activated by HBZ, but not its mutant. Dkk1 plays a major role in the development of bone lesions caused by multiple myeloma. In parallel with the initial findings, activation of Dkk1 expression by HBZ was abrogated by siRNA-mediated knockdown of p300/CBP or by a truncated form of p300 containing the KIX domain. Among HTLV-1-infected T-cell lines tested, the detection of Dkk1 mRNA partially correlated with a threshold level of HBZ mRNA. In addition, an uninfected and an HTLV-1-infected T-cell line transfected with an HBZ expression vector exhibited *de novo *and increased DKK1 transcription, respectively. In contrast to HBZ, The HTLV-1 Tax protein repressed Dkk1 expression.

**Conclusions:**

These data indicate that HBZ activates Dkk1 expression through its interaction with p300/CBP. However, this effect is limited in HTLV-1-infected T-cell lines, which in part, may be due to suppression of Dkk1 expression by Tax. Consequently, the ability of HBZ to regulate expression of Dkk1 and possibly other cellular genes may only be significant during late stages of ATL, when Tax expression is repressed.

## Background

Human T-cell leukemia virus type 1 is the etiologic agent of adult T-cell leukemia (ATL) [1-3]. ATL is characterized by uncontrolled proliferation of virally-infected CD4 + T-cells that are capable of invading the skin and other organs [4]. Patients diagnosed with the most severe forms of ATL, the acute and lymphoma subtypes, exhibit a mean survival time of less than one year and are ultimately unresponsive to chemotherapy [5]. These late stages of ATL are often associated with elevated serum calcium concentrations and sometimes with the development of lytic bone lesions, with the former condition frequently serving as the underlying cause of patient mortality [6-9]. Bone involvement of ATL is linked to a marked increase in the population of active osteoclasts [7,9]. This change is believed to shift the balance between bone resorption by these cells and matrix formation by osteoblasts in favor of overall bone loss.

ATL cells from patients and HTLV-1-infected T-cells maintained in culture have been reported to overexpress and secrete specific cytokines and other effectors that stimulate the proliferation of osteoclast precursors and/or promote osteoclast differentiation, such as IL-1, IL-6, TGF-β, TNF-α and PTH-rP [10-15]. In addition, ATL cells from patients with hypercalcemia have been found to overexpress RANKL on their membrane surface potentially through increased paracrine signaling by MIP-1α, which is also highly expressed by these cells [16,17]. Normal expression of RANKL on the surface of osteoblasts plays an essential positive role in multiple transition stages of osteoclast differentiation [18]. Possibly supporting the role of RANKL in ATL, HTLV-1-infected T-cells were recently reported to downregulate the expression of osteoprotegrin (OPG) in co-cultured osteoblast precursors [19]. OPG is secreted by osteoblasts and serves as a decoy receptor for RANKL and competitively inhibits RANKL-mediated osteoclastogenesis [20,21]. OPG may also be neutralized by cross-reactive antibodies produced against the viral envelop glycoprotein, gp46 [22].

Certain cytokines implicated in promoting hypercalcemia and lytic bone lesions in ATL patients are believed to contribute to similar pathological effects associated with another hematological malignancy, multiple myeloma (MM; [23]). In addition to these cytokines, accumulating evidence indicates that the secreted inhibitor of the Wnt signaling pathway, Dickkopf-1 (Dkk1), may represent one of the central mediators of bone resorption due to MM [24]. The canonical Wnt signaling pathway is activated by the association of secreted Wnt proteins with certain receptors within the Frizzled (Fz) family [25]. Once associated with an Fz receptor, the Wnt protein forms an additional interaction with the low-density lipoprotein receptor-related protein 5 or 6 (LPR5/6) co-receptor [25]. Formation of this complex induces an intracellular signaling pathway that promotes the stabilization and nuclear translocation of the transcriptional regulator, β-catenin. Within the nucleus β-catenin activates gene expression through the TCF/LEF transcription factors [25]. In mesenchymal stem cells and other osteoblast precursors, this pathway activates the expression of genes involved in osteoblast differentiation and activation [24]. Dkk1 inhibits this process by binding to LRP5/6, which competitively inhibits binding by Wnt proteins [24]. Additionally, Dkk1 bound to LRP5/6 associates with the transmembrane protein Kremen 1 or Kremen 2, inducing internalization and degradation of LPR5/6 [24].

With respect to ATL, there is a limited understanding of the mechanisms responsible for inducing expression of cytokines associated with bone loss. The viral protein Tax has been implicated in some of these processes. Tax activates transcription from the HTLV-1 promoter and also deregulates expression of numerous cellular genes [26,27]. This viral protein has been reported to activate expression of IL-1α, IL-6 and PTH-rP [28-30], and certain transgenic mice expressing Tax develop hypercalcemia [31]. However, Tax is dispensable for the overexpression of IL-1β in ATL cells freshly isolated from patients and for PTH-rP expression in certain model systems [10,32,33]. Furthermore, expression of Tax is frequently abolished during late stages of ATL by deletions in the proviral genome or reversible modifications such as DNA methylation [34,35]. Therefore, although Tax may facilitate the development of hypercalcemia, it is not the singular viral factor involved in this process.

Unlike Tax, the expression of the HTLV-1 basic leucine zipper factor (HBZ) is consistently detected in ATL cells [36]. This property is due to the unique location of the HBZ gene on the negative strand of the provirus [37]. Therefore, HBZ transcription is regulated by a promoter within the 3' long terminal repeat (LTR) rather than by the 5' LTR promoter that is responsible for transcription of all other HTLV-1 genes [38,39]. Accumulating evidence indicates that HBZ plays a role in the development of ATL (reviewed in [40]). HBZ has been shown to repress viral transcription as well as to deregulate the expression of cellular genes [36,37,41-43]. Although the viral protein mediates many of these processes, including repression of HTLV-1 transcription, the HBZ mRNA has also been reported to alter cellular gene expression [36,44]. The effects of the RNA were localized to a specific hairpin secondary structure in the 5' portion of the molecule [36].

The repression of HTLV-1 transcription by HBZ stems from two distinct domains in the viral protein. The C-terminal region of HBZ contains a leucine zipper (ZIP) domain that mediates dimerization with certain basic leucine zipper (bZIP) transcription factors [37]. Some of these cellular factors, including CREB, CREB-2, CREM, ATF1 and c-Jun, are involved in HTLV-1 transcriptional regulation. When bound by HBZ, these factors are unable to associate with the viral promoter to activate transcription [37,45,46]. This effect is due to the divergent basic region of the bZIP domain in HBZ that is not known to target a specific DNA sequence. In addition to the bZIP domain, HBZ harbors an N-terminal activation domain that contains two LXXLL motifs. These motifs mediate direct binding of HBZ to the homologous cellular coactivators CBP and p300, which specifically occurs through the KIX domain that is conserved between the coactivators [47,48]. CBP and p300 play central roles in the activation of HTLV-1 as well as cellular transcription by serving as scaffolds for other transcriptional regulators to associate with promoters and through their histone acetyltransferase activity [48]. In the context of HTLV-1 transcription, HBZ effectively displaces p300/CBP from the viral promoter [47]. This mechanism appears to be more potent than that of the bZIP domain in mediating repression of viral transcription.

To identify alterations in cellular gene expression caused by the HBZ-p300/CBP interaction, we established HeLa cell lines stably expressing HBZ or HBZ mutated in both LXXLL motifs. A preliminary comparison of the gene expression profiles between these cell lines identified *DKK1 *as a gene potentially upregulated by wild-type HBZ, but not by its mutant. We confirmed that the levels of the Dkk1 glycoprotein were higher in the culture medium from cells expressing wild-type HBZ compared to medium from cells expressing the mutant. This effect was attributed to the LXXLL motifs in HBZ, as mutations disrupting the leucine zipper and the RNA hairpin structure did not abrogate the activation of *DKK1 *transcription. Knock-down of p300/CBP by siRNA and expression of a p300 deletion mutant dramatically reduced Dkk1 levels, suggesting that the coactivators participate in this activation. In HTLV-1-infected T-cell lines, little or no Dkk1 mRNA was detected. Supplemental experiments revealed that Tax represses Dkk1 expression, which may partially account for the limited *DKK1 *expression in infected cells. Indeed, ectopic expression of HBZ was sufficient to activate *DKK1 *transcription in an HTLV-1-infected, as well as an uninfected T-cell line. Based on these observations, it is possible that HBZ activates Dkk1 at some stage of ATL. Such an event would likely contribute to the accelerated bone resorption associated with this disease.

## Methods

### Plasmids

pMACS K^k^.II and pMACS 4.1 are from Miltenyi Biotec, pcDNA3.1(-)/Myc-His is from Invitrogen, and pSG5 and pCMV-3Tag-8 are from Agilent Technologies. pcDNA-HBZ-SP1-Myc, pcDNA-HBZ-MutAD, pSG-Tax, pSG-M47 and pSG-M22 have been described [47,49,50]. pSG-K88A was constructed by PCR, amplifying Tax-K88A from CMV-K88A [51] and inserting the fragment into the EcoRI and BamHI sites of the pSG5 vector. pSG-HBZ-Myc was constructed by PCR, amplifying HBZ from pcDNA-HBZ-SP1-Myc [49] and inserting the fragment into the EcoRI site of the pSG5 vector. pcDNA-HBZ-MutZIP and pcDNA-HBZ-MutHP were constructed using the QuikChange II site-directed mutagenesis kit (Agilent Technologies) as described by the manufacturer to produce L168A/L182A amino acid, and C9G/T10A/C11G/A12T/G15T nucleotide substitutions, respectively. pCMV-p300_1-300_-Flag and pCMV-p300_1-700_-Flag were constructed by PCR, amplifying p300 fragments from pCMVb-p300-HA (Addgene, plasmid 10718) and cloning the fragments into pCMV-3Tag-8 at the BamHI site. pSG5-THU was constructed by inserting fragments of the *HBZ *and *UBE2D2 *genes into the BglII and XbaI sites, respectively, of pSG-Tax. Primers 5'-GAAGATCTCATCGCCTCCAGCCTCCCCT and 5'-GAAGATCTGAGCAGGAGCGCCGTGAGCGCAAG, with inserted 5' BglII sites were used to PCR amplify the *HBZ *fragment from pcDNA-HBZ-SP1-Myc [49]. Primers GCTCTAGATGCCTGAGATTGCTCGGATCTACA and GCTCTAGACGTGGGCTCATAGAAAGCAGTCAA with inserted 5' XbaI sites were used to amplify the *UBE2D2 *fragment from cDNA.

### Cell culture and transfection

HeLa cells were cultured in Dulbecco's modified Eagle's medium (DMEM) supplemented with 10% fetal bovine serum, 2 mM L-glutamine, 100 U/ml penicillin, and 50 μg/ml streptomycin. T-cell lines were cultured in Iscove's modified Dulbecco medium (IMDM) supplemented with 10% fetal bovine serum, 2 mM L-glutamine, and penicillin-streptomycin. IL2 (50 U/ml, Roche) was added to the culture medium for 1185 and SP cells. HBZ-expressing cell lines were established by transfecting HeLa cells with pcDNA-HBZ-SP1-Myc or MutAD [47], or pcDNA3.1 using Lipofectamine (Invitrogen), followed by selection with 0.5 mg/mL G418 beginning 48 h post-transfection. Clonal cell lines were obtained by expansion of individual cell colonies. Transfection of protein expression vectors into HeLa cells or the stable cell lines was done by electroporation with cotransfection of pMACS 4.1 and purification of transfected cells using the MACSelect system (Miltenyi Biotec) as described [52]. Transfection of Jurkat and MT-2 cells was done using a Gene Pulser Xcell (Bio-Rad) to electroporate 1.3 × 10^7 ^cells in 600-750 uL RPMI/10 mM dextrose/0.1 mM dithiothreitol and 20 ug plasmid DNA (3:1 stiochiometric ratio of the expression vector of interest to pMACS K^k^.II) per 0.4 cm cuvette. Each cell suspension was subjected to a single exponential decay pulse of 250 V/950 μF. Four cuvettes (pulses) were used per vector. Electroporated cells were cultured 48 h. Live cells were harvested by centrifugation on Ficoll-Paque PLUS (GE Healthcare) according to the manufacturer's instructions. Positively transfected cells were then purified using the MACSelect system.

### Small RNA interference

The siGENOME SMART pool M-003486-04-0005 and M-003477-02-0005 were used to knock-down p300 and CBP respectively, while the siGENOME Non-Targeting siRNA pool#1 D-001206-13-05 was used as a control (Thermo Scientific). Cells were seeded to reach ~50% confluence on the day of transfection. Cells were transfected with 25 nM of siRNA using DharmaFECT 1 siRNA transfection reagent (Thermo Scientific) according to the manufacturer's instructions. The medium was changed 24 h after transfection, and cells were cultured for an additional 48 h in serum-free medium prior to collection of the media (for Dkk1 expression) and the cells (for checking siRNA efficiency).

### Reverse transcriptase PCR

RNA was extracted from cells using TRIzol Reagent (Invitrogen) as described by the manufacturer. cDNA was synthesized using the iScript Kit (Bio-Rad) as described by the manufacturer. The DKK1a-R primer was used for cDNA synthesis with RNA from T-cell lines; random primers were used for all other RNA samples. Real-time PCR was performed using the iQ5 Multicolor Real-Time PCR System (Bio-Rad). Standard curves were generated from each PCR plate for all primer pairs on the plate using a serial dilution of an appropriate experimental sample. Samples were amplified in triplicate on each plate in 15 uL reactions containing 7.5 uL 2× Maxima SYBR Green/Fluorescein qPCR Master Mix (Fermentas) and 1 uL cDNA diluted 1:20. Data were analyzed using iQ5 Optical System Software (Bio-Rad). PCR efficiencies ranged from 83% to 120% with correlation coefficients of 0.95 to 1.0. Primers used were as follows: DKK1a-F, 5'-AGACCATTGACAACTACCAGCCGT; DKK1a-R, 5'-TCTGGAATACCCATCCAAGGTGCT; DKK1b-F, 5'-ATGCGTCACGCTATGTGCT; DKK1b-R, 5'-TTTCCTCAATTTCTCCTCGG; UBE2D2-F, 5'-TGCCTGAGATTGCTCGGATCTACA; UBE2D2-R, 5'-ACTTCTGAGTCCATTCCCGAGCTA; Tax-F, 5'-ATGGCCCACTTCCCAGGGTTTGGA; Tax-R, 5'-ACCAGTCGCCTTGTACACAGTCTC; HBZ-S1-F, 5'- TTAAACTTACCTAGACGGCGGACG; HBZ-S1-R, 5'-GCATGACACAGGCAAGCATCGAAA; ACTB-F, 5'-ACCAACTGGGACGACATGGAGAAA; ACTBR, 5'-TAGCACAGCCTGGATAGCAACGTA. The DKK1b primer pair was used for standard PCR amplification of cDNA prepared with the DKK1a-R primer. Forty and twenty nine amplification cycles for primer pairs DKK1b and UBE2D2, respectively, were used to achieve product amounts close to a linear range of amplification according to real-time PCR analysis. Relative mRNA levels of *DKK1 *and *ACTB *among experimental samples were determined using the 2^-ΔΔCT ^method [53], using *UBE2D2 *as the reference housekeeping gene. Relative copy numbers for UBE2D2, HBZ and Tax mRNA among HTLV-1-infected cell lines were determined by amplification of all samples with all three primer sets and a serial dilution of pSG-THU on the same plate and subsequent calculation of the mRNA copy number according to the pSG-THU standard curve.

### Detection of proteins from cellular lysates

Cellular lysates were prepared as described [54]. Amounts of total protein from lysates indicated in the figure legends were resolved by SDS-PAGE and analyzed by Western blot as described [54]. Primary antibodies used for protein detection were as follows: mouse anti-Myc (05-724) purchased from Millipore, mouse anti-actin (MAB1501R) purchased from Chemicon International, mouse anti-Flag M2 (F3165) purchased from Sigma-Aldrich, and rabbit anti-p300 (sc-584) and anti-CBP (sc-369) purchased from Santa Cruz Biotechnology. The Tax monoclonal antibody (hybridoma 168B17-46-92) was obtained from the NIH AIDS Research and Reference Reagent Program.

### Detection of Dkk1 in culture medium

**Figure 1:**
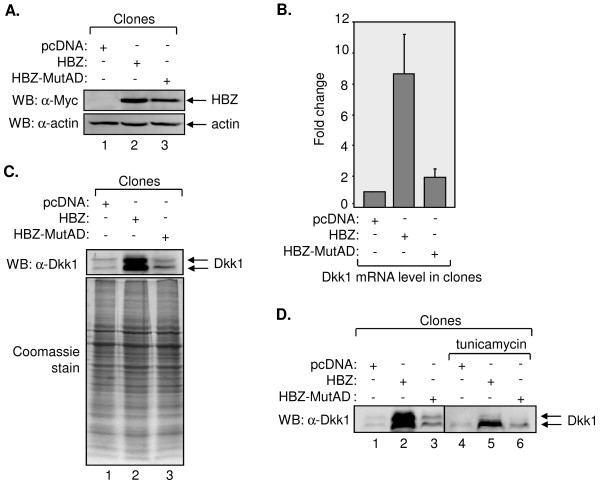
**Increased expression of Dkk1 by HBZ**. (A) Expression of HBZ wt and HBZ-MutAD in the stable cell lines. Total cellular lysates (50 μg) were subjected to Western blot analysis using antibodies directed against Myc (C-terminal epitope tag on HBZ) and β actin, as indicated. (B) Levels of Dkk1 mRNA in cells expressing HBZ wt, HBZ-MutAD, or carrying the empty vector. Levels of Dkk1 mRNA were normalized to UBE2D2 mRNA following quantitative real-time PCR of reverse transcribed total cellular RNA. The graph shows data from three independent RNA extractions, with Dkk1 mRNA levels shown relative to values obtained from cells containing the pcDNA3.1 empty vector (set to 1). (C) Levels of Dkk1 protein in the culture medium from cells expressing HBZ wt, HBZ-MutAD, or carrying the empty vector. Acid-precipitated proteins from culture media of indicated cell lines were resolved by SDS-PAGE and subjected to Western blot analysis using an antibody directed against Dkk1 (upper panel) or stained with Coomassie blue (lower panel). (D) Inhibition of Dkk1 glycosylation in cells expressing HBZ wt, HBZ-MutAD, or carrying the empty vector. The indicated cell lines were treated with DMSO (vehicle) or tunicamycin, as denoted, and acid-precipitated proteins from the culture media were analyzed by Western blot using an antibody directed against Dkk1.

Equal quantities of HeLa cells stably expressing wild-type HBZ or HBZ-MutAD, or carrying pcDNA3.1 were cultured for 24 h in serum-free medium prior to collection of the media. For Figure 1D serum-free medium was supplemented with tunicamycin (T7765, Sigma Aldrich) at a final concentration of 0.1 ug/mL. Transfected cells were cultured for 24 h in supplemented medium, purified using the MACSelect system (Miltenyi Biotec) according to the manufacturer's instructions, and equal cell quantities from each transfection group were cultured in serum-free medium for an additional 24 h prior to collection of the media. Cells and cellular debris were removed from media by centrifugation. Proteins from 0.9-1.5 mL of medium were precipitated on ice for 30 minutes in a final concentration of 10% trichloroacetic acid. Protein pellets were washed twice with ice-cold acetone and subjected to SDS-PAGE and Western blot analysis. A rabbit anti-Dkk1 (sc-25516) antibody was purchased from Santa Cruz Biotechnology. ELISAs were performed using the hDkk-1 DuoSet (R & D Systems) as described by the manufacturer. The cleared culture media were collected from transfected cells as described above, except transfected cells were not cultured in serum-free medium.

### Analysis of Dkk1 mRNA stability

**Figure 2:**
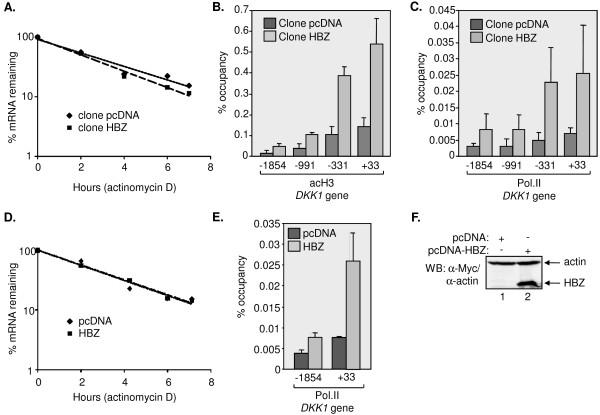
**HBZ activates Dkk1 expression at the level of transcription**. (A) Dkk1 mRNA stability in cells expressing HBZ wt or carrying the empty vector. Levels of Dkk1 mRNA were normalized to UBE2D2 mRNA following quantitative real-time PCR of reverse transcribed total cellular RNA. The graph shows relative Dkk1 mRNA levels from cells harvested at the indicated times following treatment with actinomycin D. Dkk1 mRNA levels were set to 100% at time 0 hours for both cell lines. The graph shows data averaged from two independent experiments. (B) Levels of histone H3 acetylation (acH3) at the *DKK1 *promoter in cells expressing HBZ wt or carrying the empty vector. ChIP assays were performed using an antibody directed against acH3, and relative levels of acH3 at indicated sites with respect to the mRNA start site (+ 1) were normalized to 1% of the input DNA following quantitative real-time PCR. The graph shows data averaged from three independent ChIP assays. (C) Levels of RNA polymerase II (Pol. II) enrichment at the *DKK1 *promoter in cells expression HBZ wt or carrying the empty vector, with data averaged from three independent ChIP assays. (D) Dkk1 mRNA stability in cells transiently transfected with HBZ wt or the empty vector, with data averaged from two independent transfection experiments. (E) Levels of Pol. II enrichment at the *DKK1 *promoter in cells transiently transfected with HBZ wt or the empty vector, with data averaged from two independent ChIP assays. (F) Western blot analysis of HBZ wt expression after transfection.

Clonal cells, or cells transfected and enriched using the MACSelect system (see transfection section), were plated (1.6 × 10^6^) on 6 cm plates and were cultured overnight prior to replacing normal medium with medium containing a final concentration of 0.2 ug/mL actinomycin D (A9415, Sigma Aldrich). Cells were harvested at post-treatment times indicated in Figures 2A and 2D, and processed for reverse transcriptase PCR analysis as described above. Data analysis was done as described [55].

### Chromatin immunoprecipitation (ChIP) and real-time PCR analysis of ChIP DNA

Clonal cells, or cells transfected and enriched using the MACSelect system (see transfection section), were used. For each antibody, 250 μg of formaldehyde-crosslinked chromatin was diluted to 1 mL with ChIP dilution buffer [56] and then divided into 10 and 990 μL for the input and immunoprecipitation, respectively. Other than this step, ChIP assays were performed as described [56]. Antibodies against acetyl-H3 (06-559) and RNA polymerase II (sc-9001) were purchased from Millipore and Santa Cruz, respectively. Purified input and ChIP DNA samples were suspended in 66 μL water. Real-time PCR amplification of ChIP samples was performed using the same system described above with 2.5 μL sample DNA per 15 μL reaction. PCR efficiencies and correlation coefficients ranged from 85%-110% and 0.99-1.0, respectively. Primers used were as follows: DKK1-1853F, 5'-TGGAATTTGGGATGGGAAGGACAC; DKK1-1854R, 5'-CACCACCAAGTAAAGCCAGTGACA; DKK1-991F, 5'-CATTCGGAAGCGTTGCGATGTGAT; DKK1-991R, 5'-ACTTGATTAGGCAGACGCGTGAGA; DKK1-331F, 5'-ACTTGTGTGCACAGTCAGCGAGTA; DKK1-331R, 5'-TTAATAAATGCAGGCGGCAGCAGG; DKK1 + 33F, 5'-AAATCCCATCCCGGCTTTGTTGTC; DKK1 + 33R, 5'-TCTCAGAAGGACTCAAGAGGGAGA. Protein- and modification-enrichment with each amplicon was quantified relative to the input as described [57].

## Results

### Wild-type HBZ, but not HBZ-MutAD, increases the level of Dkk1 expression

**Figure 3:**
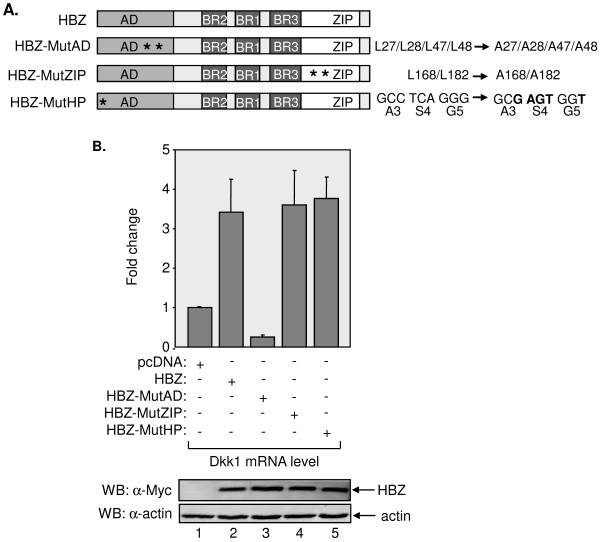
**The two LXXLL motifs in the N-terminal activation domain of HBZ are required for activation of Dkk1 expression**. (A) A schematic representation of domains in HBZ and locations of mutations that were tested. AD denotes activation domain. BR1, 2 and 3 denote basic region 1, 2 and 3, respectively. ZIP denotes leucine zipper. (B) Levels of Dkk1 mRNA in cells transfected with the indicated expression vectors. Levels of Dkk1 mRNA were normalized to UBE2D2 mRNA following quantitative real-time PCR of reverse transcribed total cellular RNA. The graph shows data from three or more independent transfections, with Dkk1 mRNA levels shown relative to values obtained from cells transfected with the pcDNA3.1 empty vector (set to 1). Lower panels show a Western blot analysis of cellular lysates (40 μg) prepared from one set of transfected cells. The membrane was probed with antibodies against Myc and β actin, as indicated.

We previously characterized an interaction between HBZ and the cellular coactivators p300 and CBP that contributes to HBZ-mediated repression of HTLV-1 transcription [47]. In that study, binding of HBZ to p300/CBP was substantially diminished by LL to AA amino acid substitutions in two LXXLL motifs located within the activation domain of the viral protein. Based on this defect of the HBZ mutant, designated HBZ-MutAD (schematically shown in Figure 3A), we were interested in determining whether the HBZ-p300/CBP interaction also affected expression of cellular genes. To begin to test this premise, HeLa cells were used to establish cell lines stably expressing wild-type HBZ (HBZ wt) or HBZ-MutAD, or cell lines carrying the empty pcDNA expression vector. HBZ wt and HBZ-MutAD, as well as other HBZ mutants used in this study are derived from the splice 1 variant of the viral protein, which is the major HBZ isoform [36,58,59]. These proteins were expressed with C-terminal Myc epitope tags to analyze their expression by Western blot (Figure 1A).

Comparison of these cells lines by preliminary gene expression microarray analysis identified *DKK1 *as a candidate gene whose expression is upregulated by HBZ wt, but unaffected by HBZ-MutAD. To corroborate a role for HBZ in regulating Dkk1 expression, we first evaluated Dkk1 mRNA levels among the cell lines using quantitative reverse transcriptase PCR (qRT-PCR). For this analysis, UBE2D2 was used as the housekeeping gene. Compared to cells containing the empty expression vector, Dkk1 mRNA was elevated more than eight-fold in HBZ wt-expressing cells, but only slightly elevated in cells expressing HBZ-MutAD (Figure 1B). Given that Dkk1 is a secreted protein, we analyzed its levels in the culture medium from each of the three cell lines by Western blot. In agreement with the observed changes in mRNA levels, Dkk1 was found to be more abundant in the medium from cells expressing HBZ wt than in media from the other cell lines (Figure 1C, upper panel). Comparative levels of total protein secreted from the cells are shown by a Coomassie-stained protein gel (Figure 1C, lower panel). Because Dkk1 is glycosylated, it was detected as a doublet, and treatment of cells with the glycosylation inhibitor, tunicamycin, reduced the detection of the upper band (Figure 1D).

### HBZ regulates Dkk1 expression at the level of transcription

HBZ is known to localize to the nucleus and directly affect activities of multiple transcriptional regulators, suggesting that it regulates Dkk1 expression at the level of transcription. However, in reporter assays we found that HBZ did not affect transcription from a region of the *DKK1 *promoter extending from -1037 to + 163 with respect to the transcription start site (data not show)[60]. This result was obtained using the reporter plasmid in a transiently transfected or a chromosomally integrated context. Similar negative results were obtained when the *DKK1 *promoter region under analysis was extended to -2034 (data not shown). Based on these data, it was possible that the HBZ-mediated increase in Dkk1 was due to stabilization of the mRNA. To test this hypothesis, we treated HeLa cells stably expressing HBZ wt or cells carrying the empty vector with the transcriptional inhibitor, actinomycin D. Relative Dkk1 mRNA levels from these cells were then evaluated by qRT-PCR at various time-points following the addition of the drug. The decrease in Dkk1 mRNA over time was plotted on a semi-log graph to calculate the mRNA half-life in each cell line (Figure 2A). We determined the half-lives to be 2.3 hours and 2.0 hours for cells carrying the empty vector versus cells expressing HBZ wt, respectively, suggesting that HBZ does not induce stabilization of the mRNA.

We additionally performed chromatin immunoprecipitation (ChIP) assays to test for protein marks at the *DKK1 *promoter that are frequently associated with transcriptional activation. We specifically evaluated relative levels of acetylated histone H3 (acH3) and RNA polymerase II across the promoter. Real-time PCR analysis of ChIP samples revealed that levels of acH3 and the polymerase were significantly higher at all of the promoter regions tested in cells expressing HBZ wt compared to cells with the empty vector (Figures 2B and 2C). The highest levels of enrichment for both protein marks were obtained in proximity to the transcription start site with amplicons centered at -331 and + 33 with respect to the transcription start site. These data and the mRNA stability data suggest that HBZ regulates Dkk1 expression at the level of transcription.

It was possible that increased Dkk1 expression in cells stably expressing HBZ may have arisen from the genomic integration of the expression vector or nonspecific cellular events occurring during development of the cell lines. To test this premise, we compared Dkk1 mRNA stability and RNA polymerase II-enrichment in HeLa cells transiently transfected with either the empty or HBZ wt expression vector (Figures 2D and 2E, respectively). Results from these experiments paralleled those obtained using the stable cell lines. The DKK1 mRNA half-life was estimated at 2.4 hours for each set of transfected cells. Figure 2F shows expression of HBZ in transiently tranfected cells. These data further support a role for HBZ in activating Dkk1 expression at the level of transcription.

### Activation of Dkk1 expression requires the LXXLL motifs in the activation domain of HBZ

The N-terminal activation domain encompassing the LXXLL motifs and the C-terminal bZIP domain in HBZ target separate sets of transcriptional regulators. While the activation domain interacts with p300/CBP [47], the bZIP domain interacts with a subset of cellular bZIP transcription factors [37,45,46,61,62]. Consequently, these domains may differentially affect expression of a given cellular gene. To test whether activation of *DKK1 *gene transcription was specifically mediated through the LXXLL motifs, we compared Dkk1 mRNA levels in HeLa cells transiently transfected with individual expression vectors for HBZ wt or the HBZ mutants denoted in Figure 3A. HBZ-MutZIP contains point mutations in the first leucine of the second and fourth heptad repeats of the leucine zipper domain, which renders the mutant defective for binding to c-Jun and CREB (data not shown). HBZ-MutHP contains five 5' nucleotide substitutions that disrupt the hairpin structure of the HBZ mRNA without altering the amino acid sequence. It was important to evaluate this mutant due to evidence that the RNA hairpin enhances T-cell proliferation [36]. Using qRT-PCR we found that only mutations in the LXXLL motifs abrogated activation of *DKK1 *transcription by HBZ (Figure 3B, upper panel). Expression of HBZ wt and all mutants was detectable by Western blot (Figure 3B, lower panel). These results suggest that activation of Dkk1 expression by HBZ involves p300/CBP.

### siRNA-mediated knockdown of p300/CBP inhibits Dkk1 expression

**Figure 4:**
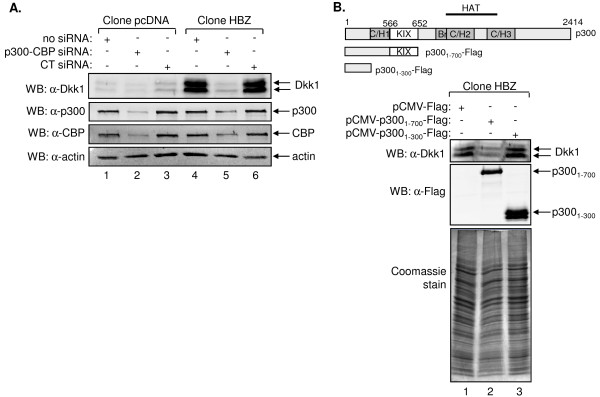
**p300/CBP function in HBZ-mediated activation of Dkk1 expression**. (A) Levels of Dkk1 in culture media following siRNA-mediated knockdown of p300/CBP. Acid-precipitated proteins from culture media (upper panel) and 25 μg of cellular lysates (lower panels) from the indicated cell lines were subjected to Western blot analysis using antibodies directed against Dkk1, p300, CBP and β actin, as indicated. (B) Levels of Dkk1 in culture media of cells co-expressing HBZ and p300 polypeptide fragments. A schematic representation of p300 domains and the fragments transfected into HBZ-expressing cells. Domains are denoted according to reference [48]. Acid-precipitated proteins from culture media (upper panel) and 20 μg of cellular lysates (middle panel) were subjected to Western blot analysis using an antibody directed against Dkk1 or the Flag epitope on transfected p300 fragments, as indicated. Acid-precipitated proteins from culture media were also resolved by SDS-PAGE and stained with Coomassie blue (lower panel).

A previous study demonstrated that p300 enhances transcriptional activation from the *DKK1 *promoter [63]. To evaluate this effect in the context of activation of Dkk1 expression by HBZ, we used siRNA to knockdown p300 and CBP expression in the cell lines carrying the empty vector or expressing HBZ wt. Western blot analysis revealed that cells transfected with siRNA molecules targeting p300 and CBP contained reduced levels of these coactivators, which was correlated with a decrease in secreted Dkk1 (Figure 4A, compare lanes 1 and 2, and lanes 4 and 5). Although apparent in both cell lines, this effect was more pronounced in cells expressing HBZ wt. No significant change in levels of Dkk1 or the coactivators was observed from cells transfected with siRNA containing scrambled sequences (Figure 4, lanes 3 and 6). It is important to note that cells remained viable over the 72 h course of this experiment [64].

We have shown that the LXXLL motifs in HBZ specifically target the KIX domain that is conserved between p300 and CBP [47]. Therefore, we expected ectopic expression of a p300 fragment containing the KIX domain to sequester HBZ from the endogenous coactivators. Such competitive interactions would be expected to abrogate HBZ-mediated activation of Dkk1 expression in a similar manner as knockdown of the coactivators. To test this hypothesis, we transfected cells stably expressing HBZ wt with an expression vector for an N-terminal fragment of p300 (aa 1-700) that contains the KIX domain (aa 566-652 for p300 [65]; pCMV-p300_1-700_-Flag). We separately transfected these cells with a shorter fragment of p300 (aa 1-300) that lacks the KIX domain. These constructs are schematically shown in Figure 4B. Western blot analysis of culture media showed that less Dkk1 was secreted from cells expressing p300_1-700 _compared to cells transfected with the empty expression vector or cells expressing p300_1-300 _(Figure 4B, upper panel). The p300 deletion mutants each contained an N-terminal Flag epitope tag for Western blot analysis of their expression (Figure 4B, middle panel). Comparative levels of total protein secreted from the cells are shown by a Coomassie-stained protein gel (Figure 4B, lower panel). These results corroborate a positive role for p300/CBP in regulating *DKK1 *gene expression and suggest that the HBZ-KIX domain interaction is important for transcriptional activation of this gene.

### Tax opposes HBZ-mediated activation of Dkk1 expression

**Figure 5:**
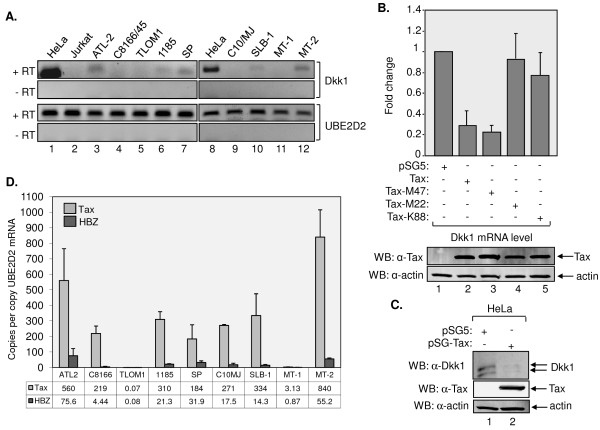
**Tax opposes HBZ-mediated activation of Dkk1 expression**. (A) Dkk1 expression in uninfected and HTLV-1-infected T-cell lines. Levels of Dkk1 and UBE2D2 mRNA from indicated cell lines were analyzed by RT-PCR. + RT and -RT denote cDNA synthesis with and without reverse transcriptase, respectively. (B) Levels of Dkk1 mRNA in cells transfected with the indicated expression vectors. Values were determined as described in the legend of Figure 1B, using data from at least three independent transfections. Lower panels show a Western blot analysis of Tax and β actin from cellular lysates (40 μg) prepared from one set of transfected cells. (C) Levels of Dkk1 in culture media from cells transfected with pSG5 or Tax. Acid-precipitated proteins from culture media (upper panel) and 30 μg of cellular lysates (lower panels) were subjected to western blot analysis. (D) Levels of HBZ and Tax expression in HTLV-1-infected T-cell lines. HBZ and Tax mRNA copy numbers were normalized to the number of UBE2D2 mRNA copies following quantitative real-time PCR and construction of a standard curve for mRNA copy number determined by amplification of 10-fold serial dilutions of the pSG-THU plasmid that contains the Tax, HBZ and UBE2D2 amplification targets. The graph shows data from at least two independent RNA extractions from each cell line. The average mRNA copy numbers for Tax and HBZ relative to UBE2D2 are indicated below each cell line.

Dkk1 is not normally expressed in T-cells [66]. To test for its expression in HTLV-1-infected T-cells, we performed a standard RT-PCR analysis using a panel of HTLV-1-infected T-cell lines. To detect low levels of Dkk1 mRNA, we prepared cDNA using a Dkk1 mRNA-specific primer. Unlike HeLa cells, little to no Dkk1 mRNA was detected in the infected cells (Figure 5A). Weak expression was observed in the HTLV-1-infected T-cell lines 1185, SP, MT-2 and SLB-1. Weak expression was also observed in the ATL cell line, ATL-2. No Dkk1 mRNA was detected from the HTLV-1-infected cell lines C10MJ and C8166, from the ATL cell lines MT-1 and TL-OmI, or from uninfected Jurkat T-cells. Because Dkk1 mRNA levels were below the quantitative range of real-time PCR, qRT-PCR was not used for this analysis.

In addition to HBZ, the viral transcription factor Tax functions to deregulate cellular gene expression and is strongly expressed in most HTLV-1-infected cell lines. Tax has specifically been shown to upregulate transcription through β-catenin, a pathway inhibited by Dkk1 [67]. Consequently, we tested whether Tax repressed *DKK1 *gene expression in HeLa cells transfected with an expression vector for wild-type Tax or individual Tax mutant expression vectors, including M47, M22 and K88A. M47 is defective for Tax-mediated transcriptional activation through CREB and SRF [68,69], M22 is defective for Tax-mediated activation of NF-KB signaling [68], and K88A is deficient for binding to the KIX domain of p300/CBP [51]. Using qRT-PCR, we found that Tax and M47 reduced the level of Dkk1 mRNA more than 3-fold, while M22 and K88A did not significantly affect expression (Figure 5B). This effect paralleled a reduction in Dkk1 in the culture medium of cells expressing Tax compared to that of cells transfected with the empty vector (Figure 5C). Based on the opposing roles of HBZ and Tax on Dkk1 expression, we quantified their mRNA levels in the HTLV-1-infected T-cell lines with respect to UBE2D2 mRNA levels using qRT-PCR. The UBE2D2 housekeeping gene exhibits stable expression in T-cells [70]. With the exception of TL-OmI, the expression of Tax mRNA was higher than that of HBZ in the cell lines (Figure 5D). The average ratio of HBZ to Tax among the cell lines tested was 0.23 ± 0.38, which is 10-fold greater than the ratio defined by Usui *et al*. [71]. This variation may reflect the different cell lines analyzed; however, the overall trend is the same with cell lines containing substantially higher levels of Tax mRNA compared to HBZ mRNA. TL-OmI cells exhibited the lowest Tax mRNA signal as expected, since these cells harbor a provirus that is transcriptionally dormant due to methylation of the 5' LTR [72]. TL-OmI also exhibited a similar low level of HBZ mRNA consistent with a previous report in which quantitative PCR was used to measure HBZ mRNA levels [73]. The highest levels of HBZ mRNA were found in ATL-2, 1185, SP, MT-2, C10MJ and SLB-1 cells. With the exception of C10MJ, these cell lines exhibited detectable Dkk1 mRNA. MT-2 cells exhibited the highest level of Tax expression. However, most Tax in MT-2 cells corresponds to a fusion protein containing a portion of Env, and only a small amount of 40 kDa Tax is present in these cells [74]. These results suggest that a threshold concentration of HBZ is required for transcriptional activation of Dkk1. It is also possible that Tax limits effects of HBZ on *DKK1 *gene expression.

**Figure 6:**
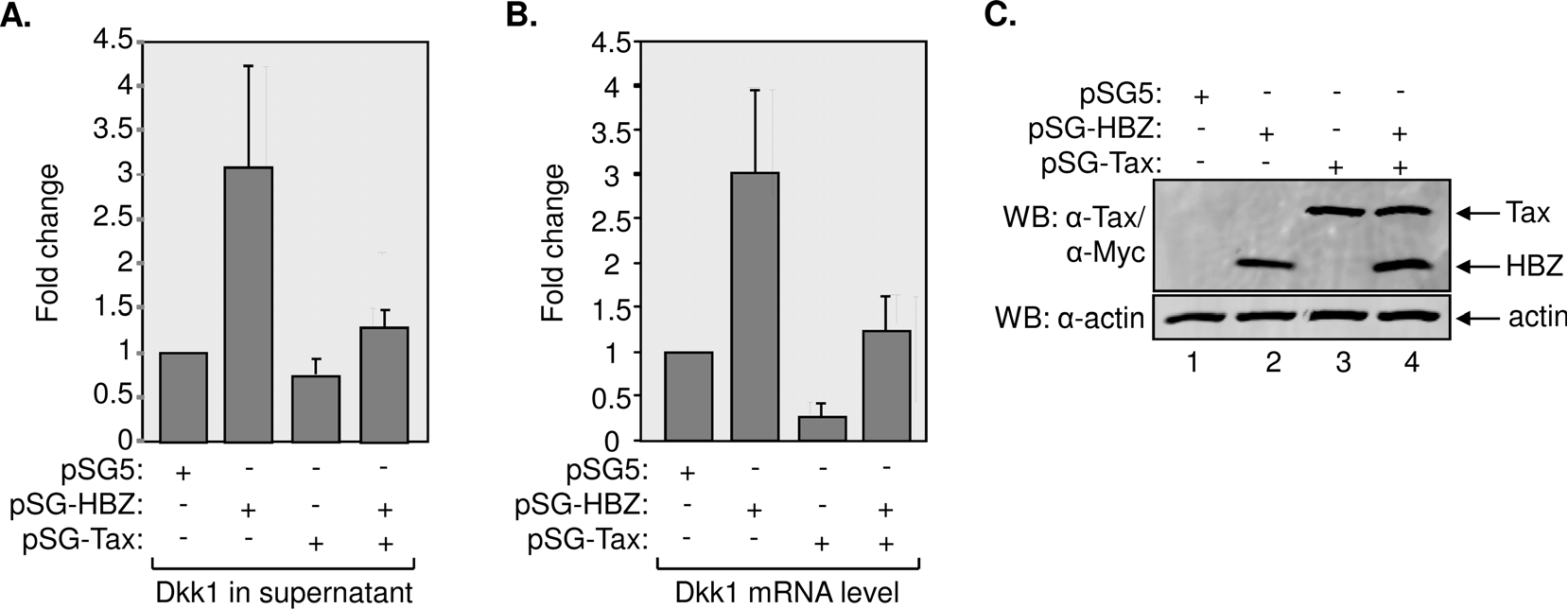
**Tax abrogates activation of Dkk1 expression by HBZ**. (A) Levels of Dkk1 in culture media from cells transfected with the indicated vectors. Dkk1 levels from culture media were detected by ELISA and normalized to the level of Dkk1 from the medium of cells transfected with the empty vector. The graph shows data from three independent transfection assays, with Dkk1 protein levels shown relative to values obtained from cells containing the pSG5 empty vector. A 1:1 stoichiometric ratio was used for cotransfection of HBZ and Tax expression vectors. (B) Levels of Dkk1 mRNA from cells transfected with the indicated vectors. Levels of Dkk1 mRNA were normalized to UBE2D2 mRNA following quantitative real-time PCR of reverse transcribed total cellular RNA. The graph shows data from two independent experiments, with Dkk1 mRNA levels shown relative to values obtained from cells containing the pSG5 empty vector (set to 1). (C) Expression of HBZ and Tax after transfection. Total cellular lysates (25 μg) were subjected to Western blot analysis using antibodies directed against Tax, Myc (C-terminal epitope tag on HBZ) and β actin, as indicated.

To determine whether Tax abrogates the activation of Dkk1 expression by HBZ, we transiently transfected HeLa cells with HBZ and Tax expression vectors alone, or in combination, and measured levels of Dkk1 protein and mRNA (Figure 6). Levels of Dkk1 protein secreted by transfected cells were quantified from culture media by ELISA. Compared to cells transfected with the control vector, the expression of HBZ and Tax led to an increase and a decrease of Dkk1 in the culture medium, respectively (Figure 6A). Strikingly, when cells were cotransfected with both HBZ and Tax, the level of Dkk1 was comparable to that of the cells transfected with the empty vector. A similar pattern was observed for the relative levels of Dkk1 mRNA from transfected cells, as determined by qRT-PCR (Figure 6B). Figure 6C shows the expression of HBZ and Tax in transfected cells. These results indicate that each viral protein is able to counteract the regulatory function of the other on Dkk1 expression.

### HBZ activates *DKK1 *gene transcription in an uninfected and an HTLV-1-infected T-cell line

**Figure 7:**
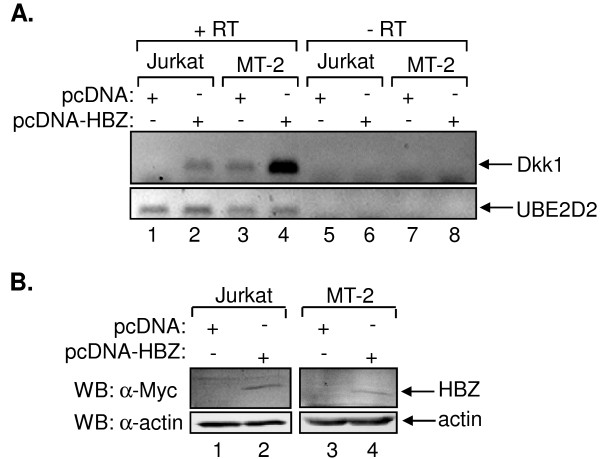
**HBZ activates Dkk1 expression in an uninfected and an HTLV-1-infected T-cell line**. (A) Dkk1 expression in uninfected and HTLV-1-infected T-cell lines. Levels of Dkk1 and UBE2D2 mRNA were analyzed by reverse transcriptase PCR. Cell lines and expression vectors are indicated. + RT and -RT denote cDNA synthesis with and without (mock synthesis) reverse transcriptase, respectively. (B) Ectopic expression of HBZ in transfected cells. Total cellular lysates from Jurkat (35 μg) and MT-2 (125 μg) cells were subjected to Western blot analysis using antibodies directed against Myc and β actin, as indicated.

We were interested in determining whether the expression of HBZ alone induces *DKK1 *gene expression in T-cells, and whether the increased abundance of HBZ would overcome possible Tax-mediated repression of this gene in HTLV-1-infected cells. To test these possibilities, we transfected Jurkat and MT-2 cells with an expression vector for HBZ and analyzed Dkk1 mRNA levels using standard RT-PCR. MT-2 cells were selected from the panel of HTLV-1-infected cells lines because they were more amenable to our method of transfection than the other cell lines. Following transfection of HBZ, we observed a significant increase in Dkk1 mRNA in MT-2 cells and *de novo *detection of Dkk1 mRNA in Jurkat cells (Figure 7A). However, Dkk1 was not detected in culture media by Western blot (data not shown). Western blot analysis confirmed HBZ expression (Figure 7B). Because Jurkat cells are uninfected, the HBZ-mediated increase in Dkk1 mRNA does not correspond to repression of HTLV-1 transcription, and therefore, the repression of Tax expression. These results indicate that HBZ is capable of inducing *DKK1 *gene transcription in T-cells and overcoming the repressive effect of Tax.

## Discussion

In the current study, we showed that HBZ activates the expression of the secreted Wnt signaling pathway inhibitor, Dkk1. The significance of this finding lies with the fact that elevated Dkk1 expression is implicated in the development of lytic bone lesions due to MM [75,76], and this pathological effect occurs more frequently in patients with advanced stages of ATL [23]. Bone lesions due to both malignancies are caused by an increase in the number of osteoclasts encompassing these lesions [23]. This alteration of the bone microenvironment disrupts the balance between matrix synthesis by mature osteoblasts and bone resorption by osteoclasts, favoring bone resorption. Because Wnt signaling is essential for the differentiation of mesenchymal stem cells into mature osteoblasts [24], Dkk1 effectively reduces the population of these cells, thereby diminishing matrix synthesis. Concomitantly, Dkk1 may favor an increase in the number of osteoblast precursors by stimulating proliferation of mesenchymal stem cells [77]. Immature osteoblasts within this population express high levels of RANKL [78], which plays a central role in osteoclastogenesis [18]. In addition to these effects, Dkk1 was recently shown to counteract Wnt3a-mediated negative and positive regulation of RANKL and OPG expression, respectively, in osteoblasts [79]. Specifically, Dkk1 increases membrane-bound RANKL on these cells and decreases extracellular concentrations of the OPG decoy receptor. Such a shift in the ratio of RANKL to OPG plays a major role in bone resorption associated with MM [80]. Elevated RANKL expression and reduced OPG expression have been reported to promote the osteoclastogenesis associated with ATL. Indeed, Nosaka *et al*. (2002) showed that elevated RANKL expression in ATL cells directly correlated with hypercalcemia [16]. Recently, HTLV-1-infected cells were found to deregulate the expression of OPG in osteoblast precursors [19]. Based on these findings, it is possible that, in some circumstances, HBZ activates Dkk1 expression, thereby indirectly facilitating changes in RANKL and OPG expression.

An HBZ-mediated increase in the level of Dkk1 may represent one of multiple possible mechanisms or contributing factors that culminate in the bone resorption associated with ATL. A number of the cytokines have been implicated in this process, but most are not consistently overexpressed in, or do not have their expression restricted to, ATL cells from patients specifically presenting with hypercalcemia and/or bone lesions [81]. However, elevated RANKL expression in ATL cells does correlate well with these pathological effects. Although autocrine signaling through MIP-1α has been reported to underlie increased RANKL expression in ATL cells [17], it is possible that secretion of Dkk1 may stimulate RANKL expression in other cell types within the bone microenvironment. The status of Dkk1 levels in ATL patients, particularly from bone aspirates, has not been reported.

As with many extracellular proteins with potential roles in bone resorption associated with ATL, Dkk1 is expected to elicit effects from cells located within the bone microenvironment. However, malignant cells are not consistently found in bone biopsies of ATL patients presenting with hypercalcemia and/or lytic bone lesions (for example see [7,9]). A possible explanation for this discrepancy may involve HTLV-1 infection of cell types other than activated CD4 + T-cells. In one study Koyanagi *et al*. (1993) identified viral DNA in CD8 + T-cells, monocytes and B-cells (in addition to CD4 + T-cells) from individuals infected with HTLV-1, including ATL patients [82]. Given that MM is a B-cell-derived malignancy, it is possible that HTLV-1 infection of B-cells and HBZ-mediated activation of Dkk1 in these cells facilitates the bone resorption process in ATL. It is also possible that HBZ produces more robust Dkk1 expression in these cell types compared to the low expression observed in T-cell lines.

HBZ-mediated activation of Dkk1 expression appears to occur through an uncommon mechanism that does not involve the promoter region immediately upstream of the transcription start site. HBZ failed to activate transcription from a reporter plasmid containing the *DKK1 *promoter both in the context of the transiently transfected and the chromosomally-integrated plasmid (data not shown). Evidence that HBZ regulates Dkk1 expression at the level of transcription is based on observations that the increase in Dkk1 mRNA by HBZ is not due to stabilization of the mRNA. Furthermore, HBZ expression coincides with increased acetylation of histone H3 over the promoter and increased RNA polymerase II abundance in proximity to the transcription start site, which frequently mark sites of transcriptional activation. Although cis elements required for HBZ-mediated activation may lie in proximity to, but outside the *DKK1 *promoter regions tested, it is also possible that HBZ functions through a distal enhancer element. Experimental evidence indicates that optimal expression of Dkk1 in mice relies on a currently uncharacterized enhancer element within a 60 kb stretch of DNA that is located 150 kb from the 3' UTR of the *DKK1 *gene [83]. Such a regulatory mechanism may help dictate levels of Dkk1 expression in human cells.

Results from this study indicate that HBZ activates *DKK1 *transcription through its interactions with the cellular coactivators, p300 and CBP. HBZ forms a stable complex with p300/CBP through binding of two LXXLL motifs in the activation domain of the viral protein to the KIX domain of the coactivators [47]. Mutating both LXXLL motifs, which severely diminishes binding to p300/CBP, rendered the viral protein incapable of activating *DKK1 *transcription. In contrast, no loss in transcriptional activation was observed using an HBZ mutant with a defect in binding certain cellular bZIP transcription factors. Furthermore, an N-terminal fragment of p300 containing the KIX domain abrogated HBZ-mediated activation of Dkk1 expression, indicative of a competitive effect by this coactivator fragment. This observation paralleled results involving siRNA-mediated knockdown of p300 and CBP.

It is currently unclear how HBZ, in conjunction with p300/CBP, modulates Dkk1 expression. Interestingly, recent evidence supports a role for p300 in the regulation of gene expression through enhancers [84]. If an enhancer is involved in regulating transcription from the *DKK1 *promoter in human as it is in mouse, HBZ and p300/CBP may be acting through such a genomic site. To date, we have not detected consistent HBZ-mediated enrichment of p300/CBP at the *DKK1 *promoter (data not shown). However, it is possible that p300, acting through an enhancer, is not intimately associated with the *DKK1 *promoter, which may limit quantification of its enrichment at the promoter. We are currently improving the ChIP assay to better detect proteins outside the immediate proximity of the DNA. Studies are also underway to test whether HBZ and p300/CBP function through an enhancer to activate Dkk1 expression.

The ability of HBZ to activate Dkk1 expression may be dependent upon the abundance of HBZ as well as on other factors regulating this gene. Indeed, with the exception of C10MJ, detection of Dkk1 mRNA appeared to require a threshold level of HBZ mRNA among the HTLV-1-infected T-cell lines that we tested. Furthermore, in one of these cell lines, additional production of HBZ from a transfected vector increased the level of Dkk1 mRNA. However, the overall level of Dkk1 expression remained relatively low in this cell line, suggesting that potent mechanisms are in place to offset HBZ-mediated activation. We found that one such mechanism involves Tax, as this viral protein repressed Dkk1 expression. Interestingly, Tax-expressing HTLV-1 cells have been reported to exhibit increased stability and nuclear localization of β-catenin [67]. Although attributed to signaling through Akt, this effect may also involve repression of Dkk1 expression.

Two Tax mutants that we tested were unable to repress Dkk1 expression: M22, which is defective for activation of NF-KB signaling [68], and K88A, which is defective for binding to the KIX domain of p300/CBP [51]. With respect to the latter mutant, it is possible that binding of Tax and HBZ to p300/CBP produce direct opposing effects on Dkk1 expression. Consequently, the ability of HBZ to regulate expression of Dkk1 and potentially other genes may only be significant following loss of Tax expression, which is an event frequently observed during progression of ATL [35].

## Competing interests

The authors declare that they have no competing interests.

## Authors' contributions

NP and IL conceived the study. NP, HG and IL performed experiments. NP and IL analyzed the data. NP wrote the paper. JMM provided key reagents and important input in the design of experiments. JMM and IL critically reviewed the manuscript. All authors read and approved the final manuscript.
